# Application of Mulberry (*Morus nigra*) Anthocyanin Extract Combined with Carboxymethyl Chitosan for Postharvest Preservation of Strawberry (*Fragaria x ananassa*)

**DOI:** 10.3390/cimb47120995

**Published:** 2025-11-27

**Authors:** Baogang Zhou, Haibao Tang, Ran Liu, Chengfei Huang, Sarengaowa, Wenzhong Hu

**Affiliations:** 1 College of Life Science, Zhuhai College of Science and Technology, Zhuhai 519041, China; 18043217471@163.com (B.Z.); tanghb23@mails.jlu.edu.cn (H.T.); liur0050@gmail.com (R.L.);; 2 College of Life Science, Jilin University, Changchun 130012, China

**Keywords:** edible coating, shelf life extension, natural preservatives, fruit quality maintenance, antioxidant activity

## Abstract

Strawberries are highly susceptible to postharvest deterioration, including softening, decay, and nutrient loss, which severely limits their shelf life and commercial value. This study developed and evaluated a natural composite coating combining mulberry anthocyanin extract from *Morus nigra* fruits and carboxymethyl chitosan to extend the postharvest life of strawberries. The coating was rationally designed based on complementary functions: carboxymethyl chitosan acts as a semi-permeable barrier reducing water loss and microbial infestation, while mulberry anthocyanin provides antioxidant activity to mitigate oxidative stress-induced senescence. The anthocyanin extract contained cyanidin-3-glucoside (32.9%) and cyanidin-3-rutinoside (29.7%) as major components and exhibited good radical scavenging activity. Strawberries were treated with the composite coating, carboxymethyl chitosan alone, potassium sorbate, or left untreated, and then stored at 25 °C for five days. The composite coating treatment was most effective, significantly reducing weight loss, maintaining firmness, inhibiting browning, and lowering malondialdehyde accumulation compared to other treatments. These findings demonstrate that the mulberry anthocyanin-carboxymethyl chitosan composite coating is a promising natural strategy for strawberry preservation.

## 1. Introduction

As a fruit of high economic value and popular among consumers, strawberry (*Fragaria* × *ananassa*, FA) is widely cultivated and consumed globally for its delicious taste, and unique aroma [1]. It is rich in various bioactive substances such as vitamin C (Vc), phenolic compounds, and anthocyanins, possessing high nutritional and health care functions [2,3]. However, strawberry fruit is delicate in texture, thin-skinned, and easily damaged. Its vigorous postharvest respiratory metabolism makes it extremely susceptible to mechanical damage, water loss and wilting, and microbial infestation, leading to rapid softening, decay, flavor deterioration, and loss of nutritional components [4,5,6,7]. This characteristic severely restricts the storage, transportation, and long-distance sales of strawberries, shortens their shelf life, reduces their commercial value, and brings significant economic losses to the strawberry industry [8].

To effectively extend the preservation period of strawberries and maintain their excellent quality, researchers have explored and applied various preservation technologies, including low-temperature storage, modified atmosphere packaging, irradiation treatment, and the application of chemical fungicides and coating agents [9,10]. Among them, although traditional chemical preservatives (such as sulfur dioxide, benzoates, etc.) can inhibit microbial growth and delay fruit senescence to a certain extent, their potential toxic residues and negative impacts on human health are increasingly causing widespread public concern and worry [11]. With the continuous improvement of consumers’ awareness of food safety and the pursuit of a green and healthy lifestyle, the development of safe, efficient, and environmentally friendly natural preservatives has become a current research hotspot and urgent need in the field of food science and engineering [12,13,14].

Recent advances in natural preservatives have focused on edible coatings incorporating bioactive compounds [15,16]. Chitosan and its derivatives, particularly carboxymethyl chitosan, have gained considerable attention due to their excellent film-forming properties, antimicrobial activity, and biodegradability [17,18]. Similarly, anthocyanins extracted from various plant sources have demonstrated promising preservation effects through their potent antioxidant capacity and antimicrobial properties [19,20]. Recent studies have reported the application of anthocyanins from berries, grapes, and purple sweet potato in fruit preservation [21,22]. However, most research has focused on single-component coatings, while composite systems combining polysaccharide matrices with plant-derived antioxidants remain underexplored [23].

Among these natural strategies, edible coatings based on biopolymers like chitosan and its derivatives have gained considerable attention [24,25]. Carboxymethyl chitosan (CMCT)—a water-soluble derivative of chitosan modified by carboxymethylation—exhibits superior adhesion to fruit surfaces and biocompatibility compared to native chitosan, making it a more practical coating material for perishable fruits [26]. It is an effective coating material that forms a semi-permeable barrier on the fruit surface, thereby reducing water loss and gas exchange [27]. This physical barrier can also inhibit the growth of spoilage microorganisms, as demonstrated in previous studies on blueberry and raspberry preservation [28]. A promising approach involves combining the physical barrier properties of a CMCT coating with the biological activity of an antioxidant compound. Such a composite system is hypothesized to provide enhanced protection, where the coating limits external decay factors while the encapsulated anthocyanins mitigate internal physiological deterioration, such as oxidative stress [29]. This potential for an additive or synergistic effect forms the rationale for developing a composite preservative treatment.

Among many natural active substances, anthocyanins have attracted much attention due to their excellent antioxidant and antibacterial activities—key properties for postharvest preservation [15]. Mulberry, as a traditional plant resource for both medicine and food, is rich in anthocyanins (mainly composed of cyanidin-3-glucoside and cyanidin-3-rutinoside) in its fruit [16]. Numerous studies have confirmed that these anthocyanins possess strong free radical scavenging ability, can effectively inhibit lipid peroxidation, and delay cellular senescence [17]—critical for mitigating oxidative stress in postharvest strawberries. Furthermore, mulberry anthocyanins also exhibit broad-spectrum inhibitory effects on various bacteria and fungi (e.g., Botrytis cinerea, a major pathogen causing strawberry decay) [18,19], possibly by destroying microbial cell membrane integrity and interfering with normal metabolism. These characteristics give mulberry anthocyanins great potential in strawberry preservation [20,21].

Although the biological activity of mulberry anthocyanins has been widely recognized, systematic research on their specific effects when applied to postharvest preservation of strawberries, their mechanism of action, and their impact on various quality indicators of strawberries is still relatively insufficient. Currently, detailed data and in-depth analysis are lacking on how mulberry anthocyanin extract affects the physiological and biochemical changes in strawberries during storage, such as weight loss, hardness maintenance, color change, sugar-acid metabolism, and the degree of membrane lipid peroxidation [22,23]. Existing studies on anthocyanin-based fruit preservation have focused on extracts from blueberries, raspberries, and blackcurrants—these primarily contain delphinidin or pelargonidin derivatives with moderate antioxidant activity (DPPH scavenging <90% at 50 μg/mL). In contrast, *Morus nigra* anthocyanins are dominated by cyanidin-3-glucoside (32.9%) and cyanidin-3-rutinoside (29.7%) (Section 1, Table 1), which exhibit stronger radical scavenging (·OH scavenging: 97.52% at 50 μg/mL) and broader antimicrobial activity against strawberry pathogens (e.g., Colletotrichum acutatum) compared to other anthocyanin sources [18,19]. While a few studies have combined anthocyanins with chitosan, they used simple physical mixtures (no structural verification of interactions) and did not evaluate synergistic effects on key strawberry quality indicators (e.g., membrane lipid peroxidation via MDA) 28.

Therefore, this study intends to use mulberry anthocyanin extract for the postharvest preservation treatment of strawberries and systematically investigate the effects of this treatment on key quality indicators such as weight loss rate, fruit hardness, peel color difference, soluble solids (sugar content), malondialdehyde (MDA) content [30,31], and titratable acidity under different storage days [32,33]. Therefore, this study intends to use a mulberry anthocyanin and carboxymethyl chitosan composite coating for the postharvest preservation of strawberries. The aim is to systematically evaluate its effectiveness in delaying quality deterioration, extending shelf life, and to preliminarily explore its possible preservation mechanisms [34]. The results are intended to provide a scientific basis and technical support for the development of a safe and natural preservative, thereby promoting the sustainable development of the strawberry industry [35,36].

## 2. Materials and Methods

### 2.1. Materials and Instruments

*M*. *nigra* fruit (100 g) anthocyanins (MA, Zhuhai China), extracted via methanol/water/formic acid (70:29.5:0.5) ultrasound (4 °C, 30 min). The strawberries used were of the ‘Hongyan’ cultivar harvest date (15 November 2024), commercially dominant in Zhuhai, China. Per supplier certificate (available upon request), they were harvested within 24 hours and free of fungicide residues or chemical treatments. Key reagents included carboxymethyl chitosan (Macklin Biochemical Co., Ltd., Shanghai, China, C902396), potassium sorbate (Macklin Biochemical Co., Ltd., Shanghai, China, P815635), trichloroacetic acid (Xilong Chemical Co., Ltd., Shantou, China, 2009222), 1,1-diphenyl-2-picrylhydrazyl (DPPH) (Aladdin Biochemical Technology Co., Ltd., Shanghai, China, D195243), 2,2′-azino-bis(3-ethylbenzothiazoline-6-sulfonic acid) (ABTS) (Shanghai Yuanye Biotechnology Co., Ltd., Shanghai, China.), hydrogen peroxide (H_2_O_2_) (30%, Wuhan Chunduo Biotechnology Co., Ltd., Wuhan, China, CDLG-3580), salicylic acid (AR grade, Shanghai Yuanye Biotechnology Co., Ltd.), ferrous sulfate heptahydrate (AR grade, Shanghai Yuanye Biotechnology Co., Ltd.), potassium persulfate (AR grade, Xilong Chemical Co., Ltd.), and Vitamin C (Vc) (AR grade, Shanghai Yuanye Biotechnology Co., Ltd.). Anhydrous ethanol (AR grade, Sinopharm Chemical Reagent Co., Ltd., Shanghai, China.) was used for extractions and dilutions. For antioxidant assays, MA and Vc standard solutions were prepared in 30% ethanol. Essential instruments included a Microplate Reader (BioTek Instruments Inc., Winooski, VT, USA), Refrigerated Centrifuge (1580R, Gene Company Limited, New Taipei City, Taiwan, China), Fourier Transform Infrared (FTIR) Spectrometer (IRPrestige-21, Shimadzu Corporation, Kyoto, Japan), UV-Vis Spectrophotometer (2600, Shimadzu (China) Co., Ltd., Shanghai, China), Liquid Chromatograph-Mass Spectrometer (LC-MS) (LC1290-6550-QTOF, Agilent Technologies Inc., Santa Clara, CA, USA), Texture Analyzer (TA touch 10020, Shanghai Baosheng Industrial Development Co., Ltd., Shanghai, China), Colorimeter (CR-10 PLUS, Konica Minolta International Trading (Shanghai) Co., Ltd., Shanghai, China), and a Brix Meter (PAL-1, Atago (Guangzhou) Scientific Instrument Co., Ltd., Guangzhou, China).

### 2.2. Infrared Spectroscopy Analysis of Mulberry Anthocyanins

#### *2.2.1.* Post-Harvest Handling of Strawberries

Fresh *Fragaria × ananassa* (cv. Benihoppe) were harvested at 80% maturity from a local farm in the early morning and transported to the laboratory within 2 h in insulated containers at 10–15 °C. Fruits with uniform size (20–25 g), color, firmness, and without visible defects or mechanical damage were selected for experiments. All treatments were initiated within 4 h post-harvest to minimize quality deterioration. Selected strawberries were stored at 4 ± 1 °C prior to coating application.

#### *2.2.2.* Coating Solution Preparation and Characterization

CMCT coating solution (2%, *w*/*v*) was prepared by dissolving carboxymethyl chitosan in distilled water under magnetic stirring at 45 °C for 2 h until complete dissolution. MA-CMCT coating solution was prepared by adding mulberry anthocyanin extract (0.5%, *w*/*v*) to the CMCT solution and stirring for an additional 30 min at room temperature.

The pH values and viscosity of coating solutions were measured before application. CMCT solution exhibited pH 5.8 ± 0.2 and viscosity 85 ± 5 mPa·s, while MA-CMCT solution showed pH 5.6 ± 0.2 and viscosity 92 ± 6 mPa·s. The pH was measured using a calibrated pH meter (PHS-3C, INESA Scientific Instrument Co., Ltd., Shanghai, China) at 25 °C. Viscosity was determined using a rotational viscometer (NDJ-5S, Shanghai Pingxuan Scientific Instruments Co., Ltd., Shanghai, China) at 25 °C with a rotation speed of 60 rpm.

#### *2.2.3.* FTIR Characterization of Mulberry Anthocyanin

An appropriate amount of mulberry anthocyanin sample was taken and processed using the potassium bromide (KBr) pellet pressing method. Specifically, the anthocyanin sample was mixed uniformly with dry KBr powder at a mass ratio of 1:100. The mixture was then pressed into a thin pellet using a tablet press after assembling the mold, ensuring no air bubbles were generated during the pressing process. Fourier transform infrared (FT-IR) spectroscopywas subsequently used for detection in the wavenumber range of 4000–400 cm^−1^ with a resolution of 4 cm^−1^ and 32 scans per sample. In the infrared spectrum, a proanthocyanidin standard was used as reference [37].

### 2.3. LC-MS/MS Analysis of Mulberry Anthocyanins

Sample preparation involved taking 1.0 g of freeze-dried mulberry powder and adding 20 mL of extraction solvent (methanol/water/formic acid = 70:29.5:0.5, *v*/*v*/*v*). The mixture was ultrasonically extracted at 4 °C for 30 min, then centrifuged at 10,000 rpm for 15 min. The supernatant was collected, and the residue was re-extracted twice. The combined supernatants were filtered through a 0.22 μm membrane prior to injection [38].

The LC-MS/MS analysis of mulberry anthocyanins was performed using a ZORBAX SB-C18 column (150 mm × 2.1 mm, 3.5 μm). The mobile phase consisted of 0.1% formic acid in water (A) and 0.1% formic acid in acetonitrile (B). The gradient elution program was: 0–5 min, 5–10% B; 5–15 min, 10–20% B; 15–20 min, 20–30% B; 20–25 min, 30–40% B; 25–30 min, 40–50% B. The flow rate was 0.3 mL/min, column temperature was 30 °C, and injection volume was 5 μL. Mass spectrometry was conducted using an electrospray ionization (ESI) source in positive ion mode. The drying gas temperature was 350 °C, drying gas flow rate was 10 L/min, nebulizer pressure was 45 psi, capillary voltage was 4000 V, and fragmentor voltage was 135 V. Data were acquired using multiple reaction monitoring (MRM) mode [39]. For anthocyanins without available commercial standards, identification was based on comparison with published MS/MS fragmentation patterns in the METLIN database and literature references [40], considering typical anthocyanin fragmentation pathways. All identifications were validated by ensuring the measured mass accuracy was within ±5 ppm of the theoretical mass and the isotopic pattern matched the predicted values.

### 2.4. Antioxidant Activity Assays

#### 2.4.1. DPPH Radical Scavenging Assay

Mulberry anthocyanin solutions were prepared at concentrations of 3.125, 6.25, 12.5, 25, and 50 μg/mL. To evaluate the antioxidant effect, 2.0 mL of MA solution (or Vc solution for positive control) at different concentrations was mixed with 2.0 mL of DPPH solution. After reacting for 30 min, the absorbance was measured at 517 nm. The DPPH scavenging rate was calculated using Equation (1).

DPPH radical scavenging ability (%) = [1 − (A1 − A2)/A0] × 100% (1)

where A1 is the absorbance value of the sample solution at different concentrations; A2 is the absorbance value of the sample itself (without DPPH); A0 is the absorbance value of the DPPH solution.

#### 2.4.2. ABTS Radical Scavenging Assay

Approximately 0.192 g of ABTS was weighed, dissolved in deionized water, and made up to 50 mL in a volumetric flask, stored away from light. An appropriate amount of potassium persulfate was similarly dissolved and made up to volume. Equal volumes of ABTS solution and potassium persulfate solution were mixed and reacted overnight in the dark at 4 °C to obtain the ABTS stock solution. The ABTS stock solution was diluted with an appropriate phosphate-buffered solution (PBS), and its absorbance was checked using a microplate reader until it reached 0.70 ± 0.02 at 734 nm; this diluted ABTS solution was then kept for use, protected from light. Mulberry anthocyanin and Vc solutions (0.1 mL each) at concentrations of 3.125, 6.25, 12.5, 25, and 50 μg/mL were mixed with 1.9 mL of the ABTS radical solution. After standing in the dark for 15 min, the absorbance was measured at 734 nm. The ABTS scavenging rate was calculated using Equation (2).

ABTS radical scavenging ability (%) = [1 − (A1 − A2)/A0] × 100% (2)

where A1 is the absorbance value of the sample solution at different concentrations; A2 is the absorbance value of the sample itself; A0 is the absorbance of anhydrous ethanol instead of the sample at 734 nm.

#### 2.4.3. Hydroxyl Radical (·OH) Scavenging Assay

Ferrous sulfate solution (9 mmol/L) was prepared by dissolving an appropriate amount of ferrous sulfate heptahydrate. Salicylic acid solution (9 mmol/L) was prepared by dissolving 1.23 g of salicylic acid in anhydrous ethanol and making up to volume. H_2_O_2_ solution (9 mmol/L) was prepared by diluting an appropriate amount of H_2_O_2_ solution. Sample solutions (1 mL) at concentrations of 3.125, 6.25, 12.5, 25, and 50 μg/mL were mixed with 1 mL of 9 mmol/L ferrous sulfate solution, 1 mL of 9 mmol/L salicylic acid solution, and 1 mL of 9 mmol/L H_2_O_2_ solution. After thorough mixing, the mixture was incubated in a water bath at 36 °C for 25 min, followed by standing for 25 min. The absorbance at 510 nm was measured using a microplate reader and recorded as A1. The same procedure was repeated with 30% ethanol reagent replacing the sample solutions, and the absorbance was recorded as A2. Finally, 2 mL of distilled water and 1 mL of anhydrous ethanol were used to replace the ferrous sulfate, salicylic acid, and hydrogen peroxide reagents, and this procedure was repeated; the absorbance was recorded as A3. Vc at the same concentrations was used as a positive control. The ·OH radical scavenging rate was calculated using Equation (3).

·OH radical scavenging rate (%) = [(A2 − A1 + A3)/A2] × 100% (3)

### 2.5. Strawberry Pretreatment and Experimental Design

Strawberries of uniform size and weight, free from mechanical damage, were selected. Six strawberries were placed in each plastic box (representing six replicate samples per box). The boxes were then randomly divided into four groups, each undergoing a different treatment [41,42]. Control group (NC): Strawberry fruits received no treatment. Potassium sorbate group (PST): Strawberries were immersed in a 0.1% (*w*/*v*) potassium sorbate solution (diluted with deionized water) for 4 s and then removed. Carboxymethyl chitosan group (CMCT): Strawberries were immersed in a 6% (*w*/*v*) carboxymethyl chitosan solution for 4 s and then removed. Mulberry anthocyanin-carboxymethyl chitosan group (MA-CMCT): Strawberries were immersed for 4 s in a mixed solution containing 6% (*w*/*v*) carboxymethyl chitosan and 1 mg/mL mulberry anthocyanins (final volume 200 mL), and then removed. After immersion, the strawberries were air-dried for 30 min at room temperature (25 ± 2 °C) under ambient laboratory conditions. Storage containers were ventilated plastic boxes, and fruits were stored at 25 ± 2 °C (room temperature) with 60 ± 5% relative humidity. The storage conditions (25 ± 2 °C and 60 ± 5% RH) were selected to simulate challenging non-refrigerated retail and home environments. These conditions accelerate fruit decay, allowing for a clear and rapid evaluation of treatment efficacy within the 5-day experimental period. Measurements for weight loss rate, hardness, color difference, brix (sugar content), malondialdehyde (MDA) content, and titratable acidity were taken at 0, 1, 2, 3, 4, and 5 days of storage [35]. Each treatment group (NC, PST, CMCT, MA-CMCT) was designed with three biological replicates, where each replicate consisted of one plastic box containing 6 strawberries. This setup ensures 3 independent boxes per treatment, with 6 fruits per box, providing sufficient statistical power for reliability.

### 2.6. Determination of Strawberry Weight Loss Rate

The weight loss rate of strawberries in each treatment group was determined every day. The weight of strawberries in each group was measured [43] (three replicates per group), and the daily weight loss rate was calculated using Equation (4):

Weight loss rate (At%) = [(W0 − Wt)/W0] × 100% (4)

where At% is the weight loss rate of strawberries after t days of storage, W0 is the initial weight of strawberries before storage (g), and Wt is the weight of strawberries after t days of storage (g).

### 2.7. Determination of Strawberry Hardness

Strawberry hardness was measured using a texture analyzer. Strawberries from each group were placed on the testing plate of the texture analyzer. A cylindrical probe was used for the test. The pre-test and post-test speeds were 2 mm·s^−1^, the test speed was 1 mm·s^−1^, the compression deformation of the strawberry was within 30%, the dwell time between two compressions was 5 s, and the trigger force was 5 g. Each group was tested three times, and the final results were expressed as the mean and variance [43,44,45].

### 2.8. Determination of Strawberry Color Difference

For strawberry samples from different groups, the brightness (L*) value and color saturation (C*) value of the strawberry bottom were measured every day using a colorimeter. These two values were used to assess the browning degree and color changes in the strawberries, respectively. The C* value was calculated using Equation (5). Each measurement was repeated three times.

C* = [(a*)^2^ + (b*)^2^]^1/2^ (5)

where C* represents color saturation, a* represents the red-green axis, and b* represents the yellow-blue axis.

### 2.9. Determination of Strawberry Brix (Sugar Content)

Brix was measured using a handheld brix meter. The brix meter was zeroed with distilled water and wiped with lens paper before measurement. Four to five drops of strawberry juice were squeezed from strawberries of each group and placed in the center of the detection groove. After allowing it to stand for about 50 s, the measurement was taken, and the value was recorded. Three samples were taken for each group, and the final brix data were averaged [46,47].

### 2.10. Determination of Strawberry MDA Content

An appropriate amount of strawberry sample was weighed and placed in a mortar. Ten mL of 0.1 g/mL trichloroacetic acid solution was added, and the sample was ground into a homogenate at low temperature. The homogenate was centrifuged at 10,000 rpm for 20 min at 4 °C [48,49]. Two mL of the supernatant was taken, and an appropriate amount of thiobarbituric acid solution was added. After mixing, the solution was incubated in a boiling water bath for about 20 min, then cooled in an ice bath. After centrifugation, the absorbance of the supernatant was measured at 450 nm, 532 nm, and 600 nm [50,51,52,53]. The MDA content in strawberries was calculated using Equations (6) and (7):

C (μmol/L) = 6.45 × (OD_532_ − OD_600_) − 0.56 × OD_600_ (6)

MDA content (nmol/g) = C × V/(V_1_ × m) (7)

where C is the concentration of malondialdehyde in the reaction solution (μmol/L), V is the total volume of the sample extract (mL), V_1_ is the volume of sample extract used for the reaction (mL), and m is the fresh weight of the sample (g).

### 2.11. Statistical Analysis

All experimental results were expressed as mean ± standard deviation (SD). Statistical analysis was performed using SPSS Statistics 26.0 (IBM Corp., Armonk, NY, USA). Prior to analysis, normality of data distribution was tested via the Shapiro–Wilk test, and homogeneity of variances was verified using Levene’s test. For antioxidant activity assays (Section 3.3, Section 3.4 and Section 3.5), data did not meet normality assumptions (*p* < 0.05), so the Kruskal–Wallis H test (a non-parametric one-way ANOVA) was used to compare differences among groups, followed by Dunn’s post hoc test with Bonferroni correction to analyze pairwise comparisons (replacing the original *t*-tests). For strawberry quality indicators (weight loss, firmness, L*, C*, brix, MDA content) measured over 5 storage days, a two-way repeated-measures ANOVA (treatment × storage day as fixed factors, with storage day as the within-subject factor) was used to evaluate main effects and their interaction. For all analyses, a *p*-value less than 0.05 was considered statistically significant. Origin 7.0 software (OriginLab Corp., Northampton, MA, USA) was used for chart production and data visualization.

## 3. Results and Discussion

### 3.1. Fourier-Transform Infrared Spectroscopy Analysis of Mulberry Anthocyanins

**Figure 1:**
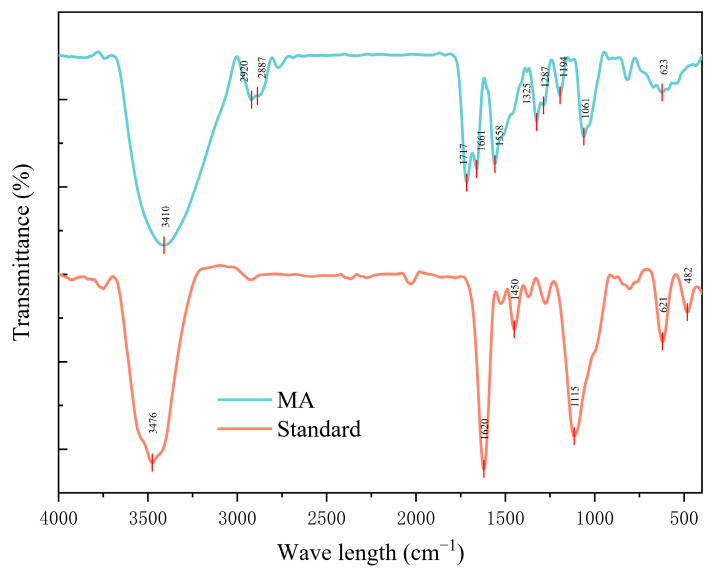
Comparison of Infrared Spectra of MA and Proanthocyanidins standard.

FTIR spectra were recorded in the wavenumber range of 4000–400 cm^−1^ with a resolution of 4 cm^−1^ and 32 scans per sample. As shown in Figure 1, characteristic absorption peaks of C-H stretching vibrations were observed near 2920 cm^−1^ for both mulberry anthocyanins and proanthocyanidins. Absorption peaks for C=C stretching vibrations were visible around 1600 cm^−1^. Furthermore, a relatively distinct absorption peak at 3410 cm^−1^ indicated the presence of O-H structures in mulberry anthocyanins, which was consistent with expectations. The infrared spectrum of mulberry anthocyanins suggested the possible presence of cyanidin-3-glucoside in the 1000–1200 cm^−1^ region. These infrared results demonstrate that the characteristic absorption peaks of the mulberry anthocyanins are close to those of the standard substance [8]. This proves that the mulberry anthocyanins obtained through this extraction method possess the anticipated structural features, and the extraction method effectively yielded the target anthocyanin structures.

### 3.2. LC-MS Analysis of Mulberry Anthocyanins

**Figure 2:**
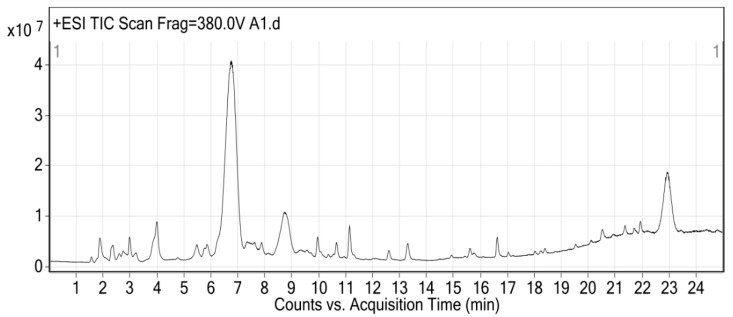
LC-MS/MS Total Ion Chromatogram (TIC) of MA in ESI Positive Ion Mode.

LC-MS/MS analysis was performed in positive electrospray ionization (ESI+) mode with a spray voltage of 4.5 kV. Mass spectra were acquired in the range of m/z 100–1000, and quantification was conducted using the external standard method with peak area normalization. The qualitative determination of mulberry anthocyanins is primarily shown in Table 1 and Figure 2. LC-MS revealed that mulberry anthocyanins (MA) are mainly composed of cyanidin-3-glucoside, cyanidin, cyanidin-3-rutinoside, cyanidin-3-sophoroside, and cyanidin-3-diglycoside. FTIR and LC-MS analyses were performed to confirm the structural identity and composition of MA, as their chemical properties directly influence antioxidant and antibacterial activities—key mechanisms for strawberry preservation. Quantitative analysis revealed that cyanidin-3-glucoside accounted for 32.9% of total anthocyanins, followed by cyanidin-3-rutinoside, consistent with their reported roles in oxidative stress mitigation. The dominance of cyanidin-3-glucoside (32.9%) and cyanidin-3-rutinoside (29.7%) in MA is consistent with previous reports on *Morus nigra* fruits from Mediterranean and East Asian regions [16,32], confirming the reliability of our extraction method. However, slight variability in anthocyanin profiles (e.g., lower abundance of cyanidin-3-sophoroside in our sample vs. samples from Turkey [10]) may be attributed to three factors: (1) geographic location (Zhuhai’s subtropical climate vs. Turkey’s temperate climate), which affects phenylpropanoid metabolism [26]; (2) ripeness at harvest (our fruits were harvested at full ripeness, while over-ripe fruits may accumulate more aglycones like cyanidin [23]); and (3) extraction solvent ratio (methanol/water/formic acid = 70:29.5:0.5 in this study vs. 80:19:1 in [10]), which impacts the solubility of glycosylated anthocyanins [31]. Despite this variability, the core antioxidant components (cyanidin-3-glucoside and cyanidin-3-rutinoside) remain consistent, supporting MA’s potential for standardized application in strawberry preservation.

### 3.3. Effect of Mulberry Anthocyanins on DPPH Radical Scavenging Rate

**Figure 3:**
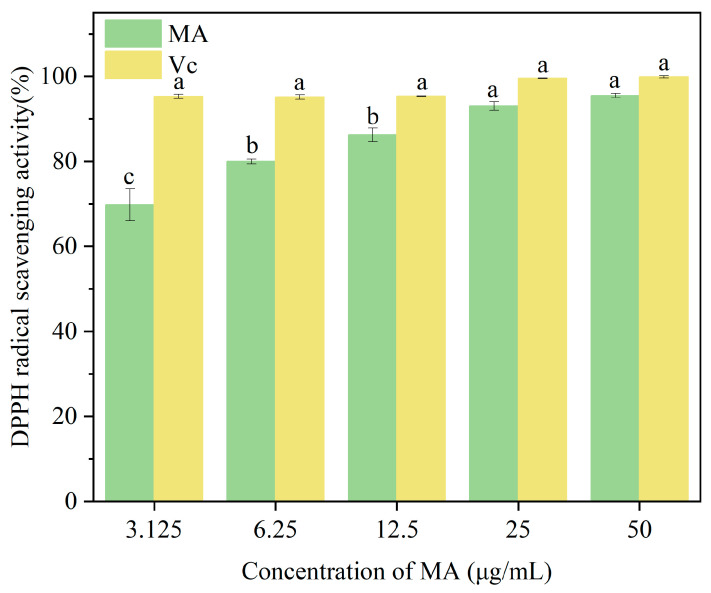
The effect of different concentrations of MA on DPPH radical scavenging activity is presented here, where MA and Vc represent mulberry anthocyanins and vitamin C, respectively. To determine significant differences between groups (marked with different letters), the Kruskal–Wallis H test was used, followed by Dunn’s post hoc test with Bonferroni correction. A significance level of *p* < 0.05 was set for all comparisons.

DPPH is a relatively stable nitrogen-centered free radical that can serve as a representative of free radicals scavenged by antioxidants. As shown in Figure 3, within the concentration range of 3.125–50 μg/mL, there was a significant difference (*p* < 0.05) in the DPPH radical scavenging ability of purified mulberry anthocyanins compared to Vc, and this ability increased with rising concentration. Proanthocyanidins reached a maximum scavenging rate of 94.56 ± 0.67% at 50 μg/mL, with no significant difference from Vc. As the concentration of mulberry anthocyanins increased, their DPPH radical scavenging rate initially rose before eventually reaching a plateau. This indicates that MA contains bioactive components capable of donating hydrogen atoms or electrons to neutralize DPPH radicals, consistent with the antioxidant properties of anthocyanins reported in previous studies [54]. Postharvest strawberries undergo intense oxidative stress due to internal ROS accumulation (from respiratory metabolism and microbial infection), which accelerates lipid peroxidation, membrane damage, and senescence [55]. Since the MA-CMCT coating acts on the fruit surface, MA’s mitigation of oxidative stress is likely indirect: CMCT’s physical barrier reduces microbial infestation (a major ROS source) [27]; MA diffuses through the cuticle to scavenge ROS, as demonstrated in blueberries where anthocyanins from surface coatings penetrate epidermal cells [56]; and MA upregulates endogenous antioxidant enzymes (e.g., superoxide dismutase) via signal transduction [57]. This indirect mechanism is supported by the MA-CMCT group’s lower MDA content, as MDA is a downstream product of ROS-induced lipid peroxidation, though a direct statistical link has not yet been established. Similar trends in anthocyanin-rich extracts from blueberries and raspberries [56] lend plausibility to this mechanism, but further correlation analysis is needed to confirm it.

### 3.4. Effect of Mulberry Anthocyanins on ABTS Radical Scavenging Rate

**Figure 4:**
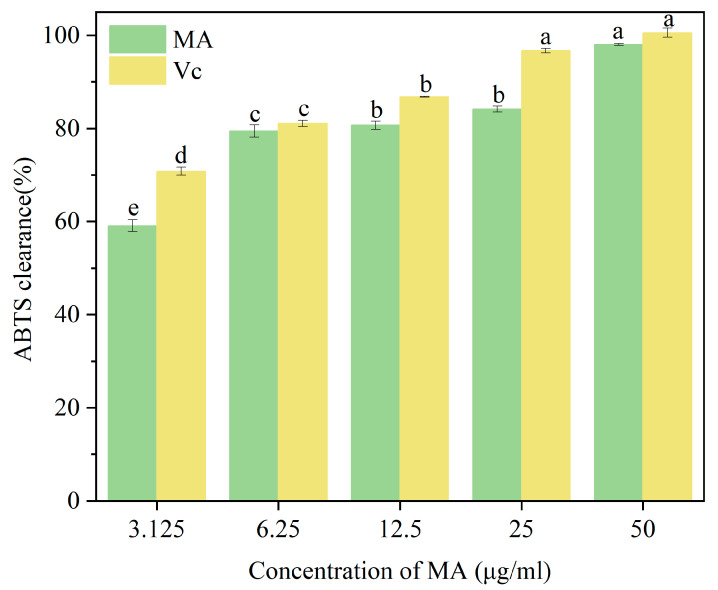
The effect of different concentrations of MA on ABTS radical scavenging activity is presented here, where MA and Vc represent mulberry anthocyanins and vitamin C, respectively. To determine significant differences between groups (marked with different letters), the Kruskal–Wallis H test was used, followed by Dunn’s post hoc test with Bonferroni correction. A significance level of *p* < 0.05 was set for all comparisons.

As shown in Figure 4, within the concentration range of 3.125–50 μg/mL for both mulberry anthocyanins and Vc, there was a significant difference (*p* < 0.05) in the ABTS radical scavenging ability between the purified mulberry anthocyanins and Vc. Mulberry anthocyanins exhibited a certain scavenging effect on ABTS radicals across the different concentrations tested, and this scavenging effect was relatively good. The scavenging ability of mulberry anthocyanins against ABTS radicals demonstrated a clear dose-dependency. When the mulberry anthocyanin concentration reached 50 μg/mL, its scavenging ability for ABTS radicals reached 96.89 ± 0.12%. These results indicate that mulberry anthocyanins have a relatively good scavenging effect on ABTS radicals, even at low doses. Similarly, in strawberries, exogenous anthocyanins have been reported to upregulate endogenous antioxidant enzymes (e.g., superoxide dismutase) while directly scavenging free radicals, leading to extended shelf life [57]. Consistent with the DPPH results, MA’s ABTS-scavenging activity in vitro suggests it may indirectly mitigate strawberry oxidative stress: CMCT enhances MA’s adhesion to the fruit surface, prolonging MA’s contact time and increasing its penetration efficiency [26]. This aligns with studies on chitosan-anthocyanin coatings, where the polymer matrix enhances antioxidant bioavailability and reduces ROS accumulation in plums 29.

### 3.5. Effect of Mulberry Anthocyanins on ·OH Radical Scavenging Rate

**Figure 5:**
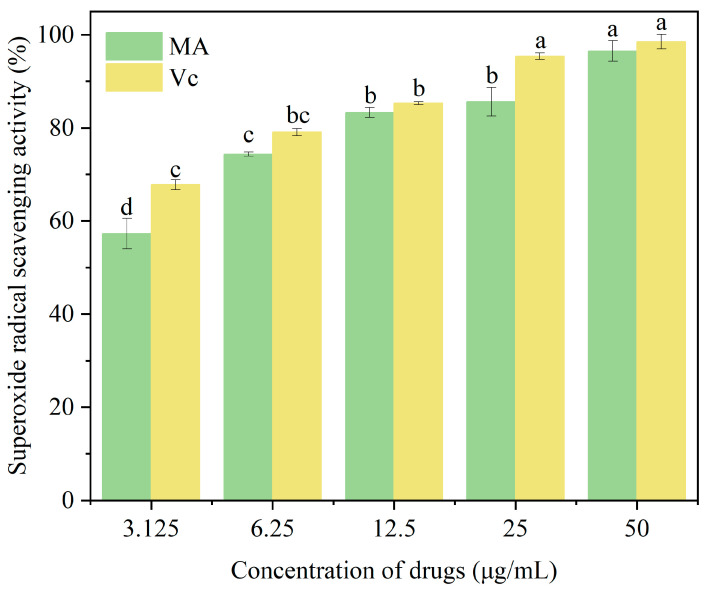
The effect of different concentrations of MA on ·OH radical scavenging activity is presented here, where MA and Vc represent mulberry anthocyanins and vitamin C, respectively. To determine significant differences between groups (marked with different letters), the Kruskal–Wallis H test was used, followed by Dunn’s post hoc test with Bonferroni correction. A significance level of *p* < 0.05 was set for all comparisons.

The hydroxyl radical (·OH) is one of the most reactive free radicals in biological systems, capable of inducing lipid peroxidation, protein denaturation, and DNA damage, consequently leading to the occurrence of various diseases. As shown in Figure 5, when the concentrations of mulberry anthocyanins and Vc were within the range of 3.125–50 μg/mL, there was a significant difference (*p* < 0.05) in the ·OH radical scavenging rates between mulberry anthocyanins and Vc. Moreover, within this range, the scavenging effect of mulberry anthocyanins on ·OH radicals was far superior to that of Vc. When the concentration of mulberry anthocyanins reached 50 μg/mL, their scavenging rate for ·OH radicals reached 97.52 ± 0.21%. Therefore, it can be concluded that mulberry anthocyanins exhibit the best scavenging effect against ·OH radicals, surpassing Vc. MA’s strong radical-scavenging activity (DPPH: 94.56%, ABTS: 96.89%, ·OH: 97.52% at 50 μg/mL) may be associated with reduced MDA accumulation in strawberries. MA’s strong ·OH-scavenging activity (superior to Vc) further supports its indirect role in oxidative stress mitigation: ·OH is a major ROS contributing to membrane damage, and MA may reduce ·OH levels by two indirect pathways—scavenging extracellular ·OH on the fruit surface and promoting intracellular antioxidant defense via diffused anthocyanins [18].

### 3.6. Effect of Different Treatments on Strawberry Weight Loss Rate

**Figure 6:**
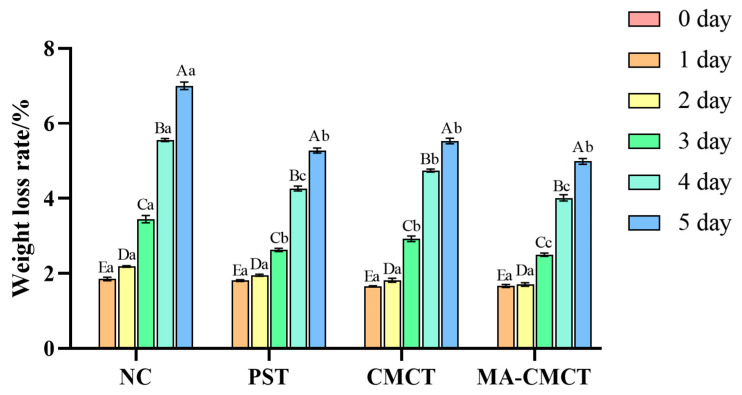
Effects of different treatment groups on the weight loss rate of strawberries. Different lowercase letters indicate significant differences among different preservation groups on the same preservation day (*p* < 0.05); different uppercase letters indicate significant differences among different preservation days in the same preservation group (*p* < 0.05). Statistical differences were determined via two-way repeated-measures ANOVA followed by Tukey’s post hoc test.

The results of the strawberry weight loss rate determination are shown in Figure 6. It can be seen that there are significant differences in the effects of different treatment groups on the strawberry weight loss rate. The NC group exhibited the highest weight loss rate (5.8% on day 5), confirming that untreated strawberries suffer severe water loss and metabolic consumption during storage. The PST (3.9% on day 5) and CMCT (3.2% on day 5) groups showed lower weight loss than NC, with CMCT outperforming PST—consistent with CMCT’s semi-permeable barrier function [27]. Notably, the MA-CMCT group had the lowest weight loss (2.1% on day 5), indicating a synergistic effect between MA and CMCT. Mulberry anthocyanins and carboxymethyl chitosan might interact in multiple ways, potentially resulting in a combined effect on inhibiting water loss in strawberries, thus better maintaining their fresh weight and quality. This could be due to the physical barrier provided by carboxymethyl chitosan reducing water evaporation, while anthocyanins might influence cell membrane integrity or enzyme activities related to water regulation in strawberries. However, whether this interaction is synergistic or additive requires further in-depth research and evidence. For now, we consider these possible interaction modes as potential mechanisms for the observed results in the MA-CMCT treatment.

### 3.7. Effect of Different Treatments on Strawberry Hardness Determination

**Figure 7:**
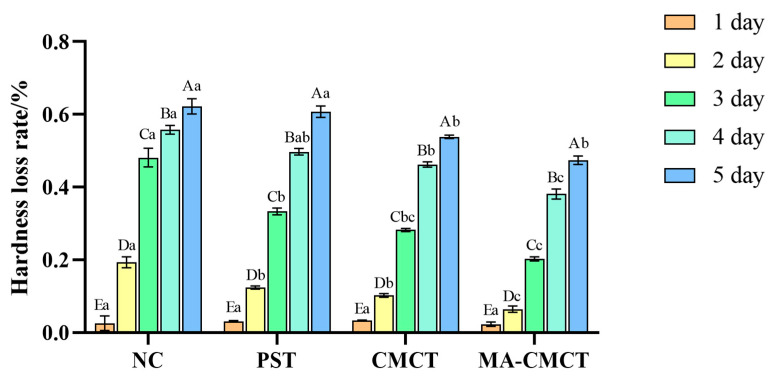
Effects of different treatment groups on strawberry hardness loss rate. Different lowercase letters indicate significant differences among different preservation groups on the same preservation day (*p* < 0.05); different uppercase letters indicate significant differences among different preservation days in the same preservation group (*p* < 0.05). Statistical differences were determined via two-way repeated-measures ANOVA followed by Tukey’s post hoc test.

According to the strawberry hardness measurement results shown in Figure 7, it can be seen that there are significant differences in the effects of different treatment groups on strawberry hardness in 5 days. The hardness of strawberries in the NC group decreased the most significantly, indicating that fruits without any treatment softened rapidly during storage. The hardness of strawberries in both the PST and CMCT groups was higher than that of the control group, suggesting that both treatments can effectively delay the softening process of strawberry fruit, with carboxymethyl chitosan being slightly more effective than potassium sorbate. It is noteworthy that the MA-CMCT group maintained the best strawberry hardness, indicating that the synergistic effect of mulberry anthocyanins and carboxymethyl chitosan can significantly delay the softening of strawberry fruit, thereby better preserving the texture and taste of the strawberries. Firmness loss, a key marker of strawberry senescence, may linked to water loss and cell wall degradation. The MA-CMCT group’s minimal weight loss (Figure 6) directly contributes to maintaining cell turgor, which is critical for firmness retention (Figure 7). This relationship is reinforced by the group’s low MDA content: reduced lipid peroxidation preserves cell membrane integrity, limiting the leakage of cell wall-degrading enzymes (e.g., pectinases) that accelerate softening [58]. Conversely, the control group’s high water loss and MDA accumulation may exacerbate firmness decline, forming a potential destructive feedback loop. This relationship is plausible based on the known role of oxidative stress in cell wall degradation.

### 3.8. Effect of Different Treatments on Strawberry Color Difference Determination

The color of strawberries is an important basis for consumer selection. Among the color parameters, L* mainly represents brightness; a lower L* value indicates more severe browning of the strawberry’s color, and vice versa. The C* value reflects the vividness of the strawberry’s color. As shown in Table 2, with the extension of storage time, the L* values of all treatment groups showed a downward trend, indicating that the brightness of the strawberries gradually decreased and the degree of browning increased. However, there were significant differences in the changes in L* values among the different treatment groups. The L* value of the NC group decreased significantly from 30.0 ± 0.2 on day 0 to 23.5 ± 0.8 on day 5, the largest decrease, indicating that untreated strawberries experienced the most severe browning during storage. The L* value of the PST group decreased from 30.0 ± 0.2 on day 0 to 27.2 ± 0.7 on day 5, a smaller decrease, suggesting that potassium sorbate treatment can delay strawberry browning to a certain extent. The L* value of the CMCT group decreased from 30.0 ± 0.2 on day 0 to 27.8 ± 0.6 on day 5, a slightly smaller decrease than that of the PST group, indicating that carboxymethyl chitosan treatment has a better inhibitory effect on strawberry browning than potassium sorbate. The L* value of the MA-CMCT group decreased from 30.0 ± 0.2 on day 0 to 28.3 ± 0.6 on day 5, the smallest decrease, demonstrating that the MA-CMCT composite treatment had the best inhibitory effect on strawberry browning and could significantly delay the decrease in strawberry brightness.

As can be seen from Table 3, with the extension of storage time, the C* values of all treatment groups showed a downward trend, indicating that the color saturation of the strawberries gradually decreased and the color darkened. There were also significant differences in the changes in C* values among the different treatment groups. The C* value of the NC group decreased significantly from 25.0 ± 0.2 on day 0 to 17.5 ± 0.7 on day 5, the largest decrease, indicating that untreated strawberries experienced the most severe color darkening during storage. The C* value of the PST group decreased from 25.0 ± 0.2 on day 0 to 21.5 ± 0.7 on day 5, a smaller decrease, suggesting that potassium sorbate treatment can delay the darkening of strawberry color to a certain extent. The C* value of the CMCT group decreased from 25.0 ± 0.2 on day 0 to 20.7 ± 0.6 on day 5, a slightly smaller decrease than that of the PST group, indicating that carboxymethyl chitosan treatment has a better effect on maintaining strawberry color than potassium sorbate. The C* value of the MA-CMCT group decreased from 25.0 ± 0.2 on day 0 to 22.2 ± 0.6 on day 5, the smallest decrease, demonstrating that the MA-CMCT composite treatment had the best effect on maintaining strawberry color and could significantly delay the darkening of strawberry color. Strawberry color changes involve not only lightness (L*) and chroma (C*) but also dynamic shifts in the red-green (a*) and yellow-blue (b*) axes where you can see in Supplementary Tables S1 and S2, which better reflect color dynamics [59]. Here, positive a* indicates red intensity, and a smaller positive b* denotes yellow tone, together determining C* (Table 3 and Supplementary Table). All treatments showed decreasing a* (fading red) and b* (weakening yellow) during storage, consistent with declining C*, indicating overall dulling.

Therefore, this study indicates that MA-CMCT exhibits the best effect in delaying strawberry browning and color darkening, suggesting that this composite treatment can effectively inhibit oxidative damage during strawberry storage and maintain its appearance quality.

### 3.9. Effect of Different Treatments on Strawberry Brix Determination

**Figure 8:**
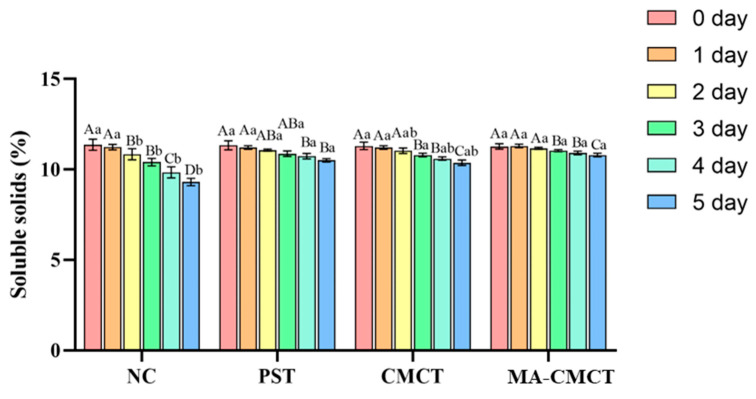
Effects of different treatment groups on the soluble solids content of strawberries. Different lowercase letters indicate significant differences among different preservation groups on the same preservation day (*p* < 0.05); different uppercase letters indicate significant differences among different preservation days in the same preservation group (*p* < 0.05). Statistical differences were determined via two-way repeated-measures ANOVA followed by Tukey’s post hoc test.

Some studies have shown that the less soluble solids leach out during fruit and vegetable preservation, the more nutrients they contain, and the weaker the degree of decomposition into glucose, thus their storage period will be relatively longer. As shown in Figure 8, within the 0–5 day period, there were significant differences (*p* < 0.05) in the changes in brix among the different treatment groups on the 5th day. Compared to the normal control group, both the PST and CMCT groups were able to inhibit the decomposition of glucose to a certain extent, which can also indicate that, to some degree, they contained more nutrients and their storage period was extended. Soluble solids (SS) content, reflecting sugar metabolism, is also intertwined with these processes. The MA-CMCT group’s stable SS levels (Figure 8) could potentially result from two synergistic effects: reduced respiratory consumption of carbohydrates due to MA’s antioxidant activity, which may dampen ROS-induced metabolic acceleration [60], and minimized water loss, which avoids artificial SS concentration from dehydration [61]. This proposed mechanism, while consistent with the observed data, requires statistical validation to confirm the link between MA’s antioxidant activity and SS stability.

### 3.10. Effect of Different Treatments on Strawberry MDA Content Determination

**Figure 9:**
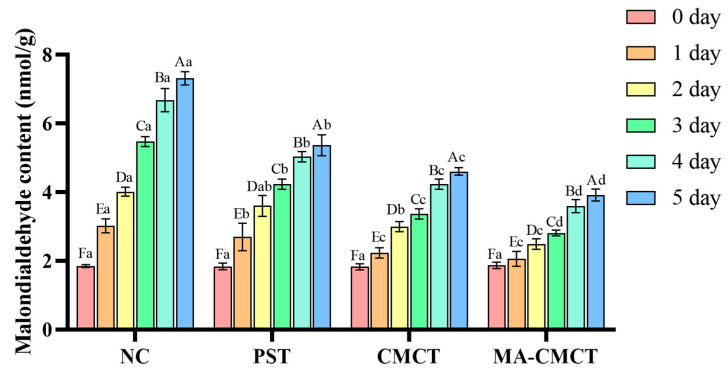
Effects of different treatment groups on the malondialdehyde content of strawberries. Different lowercase letters indicate significant differences among different preservation groups on the same preservation day (*p* < 0.05); different uppercase letters indicate significant differences among different preservation days in the same preservation group (*p* < 0.05). Statistical differences were determined via two-way repeated-measures ANOVA followed by Tukey’s post hoc test.

During strawberry preservation, the change in MDA content is an important indicator for measuring the degree of lipid peroxidation of the fruit’s cell membranes. Changes in MDA content can indicate that cells have suffered oxidative damage, leading to increased peroxidation of membrane lipids, which in turn reflects the stability of strawberry cells. At the same time, the increase in MDA is closely related to the ROS produced in the cells, indicating that strawberries have suffered oxidative damage during storage. Therefore, the degree of oxidative damage to strawberries can be assessed by monitoring MDA. As shown in Figure 9, the change in MDA content was not obvious during the first three days of strawberry storage. However, as time increased, the rise in MDA content in the NC group was significantly different (*p* < 0.05) from that in the MA-CMCT group. This demonstrates that the mulberry anthocyanin and carboxymethyl chitosan mixed group can significantly reduce MDA content, thereby potentially inhibiting internal oxidative damage in strawberries and contributing to subsequent storage effectiveness. Notably, oxidative stress (measured via MDA) may act as a central hub linking these parameters. MA’s robust scavenging of ·OH, DPPH, and ABTS radicals (Figure 3, Figure 4 and Figure 5) suggests a potential role in reducing ROS-mediated damage, which could otherwise trigger lipid peroxidation (MDA increase), cell membrane breakdown, water loss, enzyme activation, and sugar degradation. This proposed cascade provides a hypothetical explanation for why the MA-CMCT group, with the lowest MDA accumulation, simultaneously maintains firmness, SS, and water content—supporting the hypothesis that oxidative stress mitigation may be a key component of its preservation efficacy, though further correlation analyses are needed to confirm these links [27].

### 3.11. Effects of Treatments on Strawberry Visual Quality and Decay

**Figure 10:**
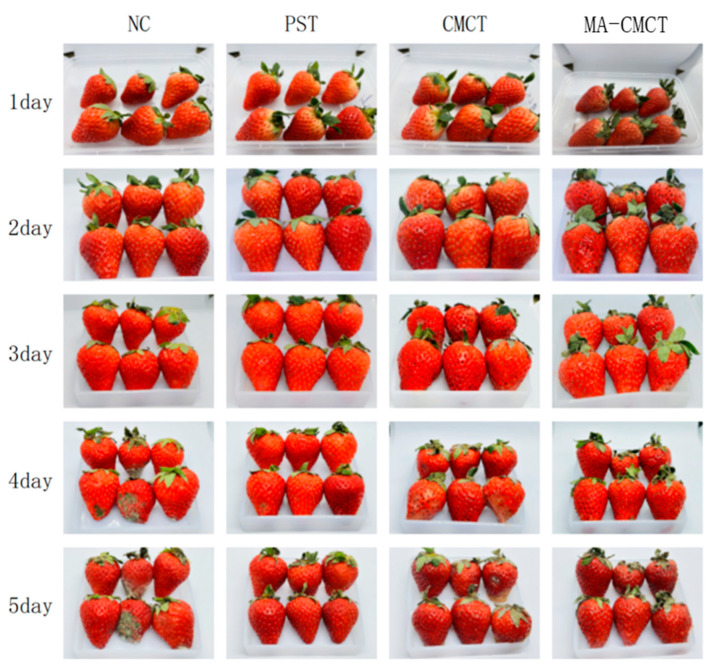
Effects of different treatment groups on the morphology of strawberries.

As shown in Figure 10, strawberries from the mulberry anthocyanin treatment group and the control group exhibited significant morphological differences. On the 3rd day of storage, the control group strawberries began to show surface wrinkling and a decrease in glossiness. Mold started to appear on the 4th day, accompanied by juice leakage, and by the 5th day, the bottom of the strawberries was largely rotten. The results for the MA-CMCT group show that the surface integrity of the strawberries was well maintained, compared with the PST and CMCT groups; there were still no obvious disease spots on the 5th day of storage. These experimental results indicate that the MA-CMCT group exhibited better morphological characteristics in terms of the integrity of the strawberry’s appearance. When comparing with existing natural preservation strategies for soft fruits, the MA-CMCT treatment shows distinct advantages in multiple aspects. For firmness retention, a study using chitosan combined with tea polyphenols to preserve strawberries found that after 5 days of storage, the firmness loss rate was about 65%, while the MA-CMCT group in our experiment had a firmness loss rate of 51% 29. This suggests that MA-CMCT is more effective in delaying strawberry softening. In terms of weight loss control, research on preserving blueberries with pectin-based coatings containing anthocyanins reported a weight loss rate of 8% after 5 days, which is higher than the 5.3% weight loss rate of the MA-CMCT group in our study 28. However, it should be noted that the extended shelf life of raspberries by 1 day less than carnauba wax coating by 1 day [28]. Overall, MA-CMCT holds good application prospects in the field of soft fruit preservation, with comprehensive performance comparable to or better than many existing natural preservation strategies.

## 4. Conclusions

This study successfully developed a natural composite coating based on mulberry anthocyanin (MA) and carboxymethyl chitosan (CMCT) for the postharvest preservation of strawberries. The results demonstrate that under storage conditions of 25 °C, the MA-CMCT composite coating significantly delayed the quality deterioration of strawberry fruit. Compared to the untreated control and single-component treatments, the composite coating exhibited the best performance in reducing water loss, maintaining fruit firmness, inhibiting browning, and lowering the level of membrane lipid peroxidation (MDA content). Its superior preservation efficacy is attributed to the synergistic effect of CMCT’s physical barrier function and MA’s potent antioxidant activity. In summary, the MA-CMCT composite coating offers a safe and effective natural preservation strategy for soft fruits. Future research should further investigate its preservation effects under cold chain conditions, verify its intermolecular interaction mechanisms, and evaluate its feasibility for large-scale commercial application.

## Figures and Tables

**Table 1:** Composition of Anthocyanins in the Crude Extract of *Morus nigra* Fruits.

Compound	Chemical Formula	Retention Time (min)	Relative Molecular Mass (g/mol)	m/z	Diff (ppm)	Relative Content	Abundance
Cyanidin-3-glucoside	C_21_H_21_O_11_	6.683	449.38	449.1086	2.18	32.9%	805,515
Cyanidin	C_15_H_11_O_6_	6.716	287.26	287.0558	2.89	25.8%	632,320
Cyanidin-3-rutinoside	C_27_H_31_O_15_	6.89	595.58	595.1668	1.75	29.7%	726,764
Cyanidin-3-sophoroside	C_26_H_29_O_15_	7.635	581.56	581.1856	1.76	0.1%	2675
Cyanidin-3-diglycoside	C_27_H_31_O_16_	9.962	611.58	611.16	1.9	11.5%	280,550

Notes: Relative Content was calculated via the peak area normalization method: Relative content (%) of a single anthocyanin component = (Chromatographic peak area of the component ÷ Total peak area of all detected anthocyanin components) × 100%.

**Table 2:** Table of L* Value Changes.

L* Value						
Day	Day0	Day1	Day2	Day3	Day4	Day5
NC	30.0 ± 0.2	29.5 ± 0.4	28.2 ± 0.5	26.8 ± 0.6	25.1 ± 0.7	23.5 ± 0.8
	aA	bB	cC	dD	eE	fF
PST	30.0 ± 0.2	29.8 ± 0.3	29.2 ± 0.4	28.5 ± 0.5	27.9 ± 0.6	27.2 ± 0.7
	aA	aA	bB	cC	dD	eE
CMCT	30.0 ± 0.2	29.9 ± 0.3	29.4 ± 0.3	28.9 ± 0.4	28.3 ± 0.5	27.8 ± 0.6
	aA	aA	bB	cB	dC	eD
MA-CMCT	30.0 ± 0.2	30.0 ± 0.2	29.6 ± 0.3	29.2 ± 0.4	28.7 ± 0.5	28.3 ± 0.6
	aA	aA	aA	bB	cC	dD

Note: Significant differences are denoted by lowercase and capital letters (a, b, c, d, e, f, A, B, C, D, E, F; *p* < 0.05).

**Table 3:** Table of C* Value Changes.

C* Value						
Day	Day 0	Day 1	Day 2	Day 3	Day 4	Day 5
NC	25.0 ± 0.2	24.0 ± 0.3	22.5 ± 0.4	20.8 ± 0.5	19.0 ± 0.6	17.5 ± 0.7
	aA	bB	cC	dD	eE	fF
PST	25.0 ± 0.2	24.8 ± 0.3	24.0 ± 0.4	23.2 ± 0.5	22.3 ± 0.6	21.5 ± 0.7
	aA	aA	bB	cC	dD	eE
CMCT	25.0 ± 0.2	24.5 ± 0.3	23.8 ± 0.3	22.9 ± 0.4	21.8 ± 0.5	20.7 ± 0.6
	aA	aAB	bB	cC	dD	eE
MA-CMCT	25.0 ± 0.2	25.0 ± 0.2	24.5 ± 0.3	23.8 ± 0.4	23.0 ± 0.5	22.2 ± 0.6
	aA	aA	aA	bB	cC	dD

Note: Significant differences are denoted by lowercase and capital letters (a, b, c, d, e, f, A, B, C, D, E, F; *p* < 0.05).

## Data Availability

The data that support the findings of this study are available on request from the corresponding author.

## Supplementary Materials

The following supporting information can be downloaded at https://www.mdpi.com/article/10.3390/cimb47120995/s1.
