# Supplementary material for: Application of Mulberry (Morus nigra) Anthocyanin Extract Combined with Carboxymethyl Chitosan for Postharvest Preservation of Strawberry (Fragaria x ananassa)
Source: Curr Issues Mol Biol. 2025 Nov 27;47(12):995. doi: 10.3390/cimb47120995 (PMC12732276; doi:10.3390/cimb47120995)
Supplement: Supplementary file 1 [file cimb-47-00995-s001.zip › cimb-3938092-supplementary.pdf]

Table S1. Table of *a** Value Changes.

*a** Value						
**Day**	**Day0**	**Day1**	**Day2**	**Day3**	**Day4**	Day5
NC	20.4 ± 0.3 aA	19.1 ± 0.4 dD	17.8 ± 0.3 dC	16.5 ± 0.5 dE	15.3 ± 0.4 dF	13.9 ± 0.6 dG
PST	20.2 ± 0.3 aA	19.8 ± 0.2 bB	19.2 ± 0.3 cB	18.6 ± 0.4 cC	17.8 ± 0.5 cD	17.2 ± 0.6 cE
CMCT	19.9 ± 0.3 aA	19.6 ± 0.2 cC	19.0 ± 0.2 cB	18.5 ± 0.3 cD	17.7 ± 0.5 cE	17.3 ± 0.4 cF
MA-CMCT	20.0 ± 0.3 aA	19.9 ± 0.2 aA	19.5 ± 0.2 bB	18.9 ± 0.4 cC	18.4 ± 0.4 cD	17.7 ± 0.4b bE

Note: Lowercase letters (a, b, c, d, e, f) indicate differences among treatments (NC, PST, CMCT, MA-CMCT) on the same storage day. Different lowercase letters in the same row mean significant differences ($p < 0.05$). Uppercase letters (A, B, C, D, E, F) represent differences among different storage days within the same treatment. Different uppercase letters in the same column imply significant differences ($p < 0.05$).

Table S2. Table of *b** Value Changes.

*b** Value						
**Day**	**Day0**	**Day1**	**Day2**	**Day3**	**Day4**	Day5
NC	15.1 ± 0.2 aA	14.3 ± 0.3 dC	13.6 ± 0.6 dD	12.4 ± 0.5 dF	11.5 ± 0.3 dG	10.4 ± 0.4 dF
PST	14.9 ± 0.2 aA	14.9 ± 0.2 bB	14.3 ± 0.4 cC	13.8 ± 0.4 cE	13.3 ± 0.5 cF	12.8 ± 0.6 cG
CMCT	15.2 ± 0.2 aA	14.6 ± 0.2 cD	14.3 ± 0.2 cC	13.7 ± 0.3 cE	13.1 ± 0.4 cF	12.4 ± 0.5 cG
MA-CMCT	14.8 ± 0.2 aA	14.9 ± 0.2 aB	14.7 ± 0.2 bA	14.3 ± 0.3 cB	13.7 ± 0.4 cE	13.3 ± 0.5b bF

Note: Lowercase letters (a, b, c, d, e, f) indicate differences among treatments (NC, PST, CMCT, MA-CMCT) on the same storage day. Different lowercase letters in the same row mean significant differences ($p < 0.05$). Uppercase letters (A, B, C, D, E, F) repre-sent differences among different storage days within the same treatment. Different uppercase letters in the same column imply significant differences ($p < 0.05$).
